# Tris(ethyl­enediammonium) bis­[(2-amino­ethyl)ammonium] bis­[bis­(μ_5_-hydrogen phosphato)penta-μ_2_-oxido-deca­oxido­penta­molybdenum(VI)] deca­hydrate

**DOI:** 10.1107/S160053681001545X

**Published:** 2010-04-30

**Authors:** Jing Lu, Hao Song, Da-Qi Wang, Mei-Ju Niu

**Affiliations:** a School of Chemistry and Chemical Engineering, Liaocheng University, Liaocheng 252059, People’s Republic of China

## Abstract

The title compound, (C_2_H_10_N_2_)_3_(C_2_H_9_N_2_)_2_[Mo_5_(HPO_4_)_2_O_15_]·10H_2_O, was prepared under hydro­thermal conditions at pH 5.0. The structure contains mono- and diprotonated ethyl­enediamine cations, [Mo_5_O_15_(HPO_4_)_2_]^4−^ anions and uncoord­in­ated water mol­ecules. The [Mo_5_O_15_(HPO_4_)_2_]^4−^ hetero­poly­oxometallate anion is made up of five MoO_6_ octa­hedra sharing an edge and forming a ring, which is closed by common corners of the terminal MoO_6_ octa­hedron. The ring is topped on both sides by two slightly distorted PO_4_ tetra­hedra, sharing three corners with three MoO_6_ octa­hedra. The terminal oxygen atoms of the PO_4_ units are protonated. Together with the anions, the water mol­ecules and the ethyl­enediammonium cations are involved in N—H⋯O and O—H⋯O hydrogen bonding, forming a three-dimensional supra­molecular network.

## Related literature

For background to polyoxometalates, see: Coronado & Gomez-Garcia (1998 ▶); Niu *et al.* (2009 ▶); Ruether *et al.* (2003 ▶). For the structure of (C_2_H_10_N_2_)_2_[Mo_5_O_15_(HPO_4_)_2_], see: Sun *et al.* (2003 ▶). For structures containing the [Mo_5_O_15_(PO_4_)_2_]^6−^ anion, see: Gong *et al.* (2006 ▶); Skibsted *et al.* (2000 ▶). For the bond-valence method, see: Brown (2002 ▶).
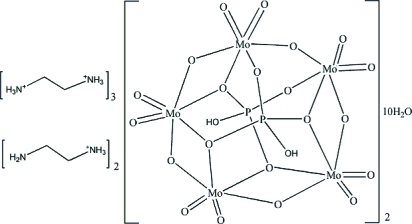


## Experimental

### 

#### Crystal data

(C_2_H_10_N_2_)_3_(C_2_H_9_N_2_)_2_[Mo_5_(HPO_4_)_2_O_15_]·10H_2_O
*M* *_r_* = 2312.06
Triclinic, 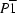

*a* = 10.0045 (11) Å
*b* = 10.6625 (12) Å
*c* = 15.1884 (19) Å
α = 87.405 (2)°
β = 73.119 (1)°
γ = 77.978 (1)°
*V* = 1516.2 (3) Å^3^
*Z* = 1
Mo *K*α radiation
μ = 2.23 mm^−1^
*T* = 298 K
0.38 × 0.34 × 0.30 mm

#### Data collection

Siemens SMART CCD area-detector diffractometer
Absorption correction: multi-scan (*SADABS*; Sheldrick, 1996 ▶) *T*_min_ = 0.485, *T*_max_ = 0.554
7582 measured reflections
5253 independent reflections
4015 reflections with *I* > 2σ(*I*)
*R*_int_ = 0.033

#### Refinement

*R*[*F* ^2^ > 2σ(*F* ^2^)] = 0.041
*wR*(*F* ^2^) = 0.114
*S* = 1.04
5253 reflections
396 parameters
5 restraints
H-atom parameters constrained
Δρ_max_ = 1.28 e Å^−3^
Δρ_min_ = −1.07 e Å^−3^

### 

Data collection: *SMART* (Siemens, 1996 ▶); cell refinement: *SAINT* (Siemens, 1996 ▶); data reduction: *SAINT*; program(s) used to solve structure: *SHELXS97* (Sheldrick, 2008 ▶); program(s) used to refine structure: *SHELXL97* (Sheldrick, 2008 ▶); molecular graphics: *SHELXTL* (Sheldrick, 2008 ▶); software used to prepare material for publication: *SHELXTL*.

## Supplementary Material

Crystal structure: contains datablocks global, I. DOI: 10.1107/S160053681001545X/wm2326sup1.cif

Structure factors: contains datablocks I. DOI: 10.1107/S160053681001545X/wm2326Isup2.hkl

Additional supplementary materials:  crystallographic information; 3D view; checkCIF report

## Figures and Tables

**Table 1:** Hydrogen-bond geometry (Å, °)

*D*—H⋯*A*	*D*—H	H⋯*A*	*D*⋯*A*	*D*—H⋯*A*
N5—H5*E*⋯O20^i^	0.90	2.66	3.075 (8)	109
N5—H5*E*⋯O28^ii^	0.90	2.01	2.796 (9)	144
N5—H5*D*⋯O10	0.89	2.45	3.069 (8)	127
N5—H5*D*⋯O6	0.89	2.01	2.846 (8)	156
N5—H5*C*⋯O21^i^	0.89	2.17	3.046 (8)	170
N4—H4*E*⋯O1^i^	0.89	1.93	2.806 (8)	167
N4—H4*D*⋯O12^iii^	0.89	2.65	3.357 (8)	137
N4—H4*D*⋯O22^iii^	0.89	2.60	3.099 (8)	116
N4—H4*D*⋯O4^iii^	0.89	2.08	2.907 (8)	155
N4—H4*C*⋯O25	0.89	1.92	2.803 (8)	171
N3—H3*D*⋯O17^i^	0.87	2.25	3.117 (8)	176
N3—H3*C*⋯O15^iv^	0.89	1.87	2.732 (7)	162
N2—H2*E*⋯O23^iii^	0.90	2.56	3.030 (8)	113
N2—H2*E*⋯O5^v^	0.90	1.84	2.699 (8)	159
N2—H2*D*⋯O20^v^	0.89	2.14	2.924 (8)	146
N2—H2*C*⋯O6^iii^	0.90	2.49	3.310 (8)	151
N2—H2*C*⋯O12^iii^	0.90	2.35	3.084 (8)	139
N1—H1*C*⋯O7^iv^	0.90	2.46	3.259 (8)	149
N1—H1*C*⋯O16^iv^	0.90	2.28	3.011 (8)	138
N1—H1*B*⋯O28^vi^	0.89	1.93	2.819 (8)	173
N1—H1*A*⋯O5^v^	0.90	1.92	2.772 (8)	159
O28—H28*B*⋯O23^vii^	0.86	2.39	3.157 (8)	149
O28—H28*A*⋯O27	0.84	2.31	2.740 (10)	112
O27—H27*B*⋯O17^i^	0.87	2.46	2.912 (10)	113
O27—H27*B*⋯O22^vii^	0.87	2.11	2.916 (10)	155
O27—H27*A*⋯O10^viii^	0.87	2.03	2.875 (9)	163
O26—H26*B*⋯O19^i^	0.84	2.40	3.064 (8)	136
O26—H26*B*⋯O17^i^	0.84	2.36	2.874 (8)	120
O26—H26*A*⋯O14	0.84	2.11	2.858 (8)	148
O25—H25*B*⋯O21^i^	0.84	1.97	2.808 (7)	170
O25—H25*B*⋯O4^i^	0.84	2.57	3.083 (7)	120
O25—H25*A*⋯O11	0.85	1.93	2.745 (7)	163
O24—H24*B*⋯O25^viii^	0.86	2.08	2.868 (9)	151
O24—H24*A*⋯O1^iv^	0.86	1.97	2.795 (8)	159
O5—H5*F*⋯O28^ii^	0.84	2.02	2.845 (8)	168
O1—H1*F*⋯N3^ix^	0.85	2.18	2.766 (8)	126

Symmetry codes: (i) 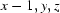; (ii) 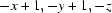; (iii) 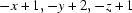; (iv) 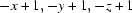; (v) 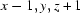; (vi) 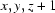; (vii) 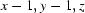; (viii) 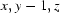; (ix) 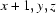.

**Fig. 1.**
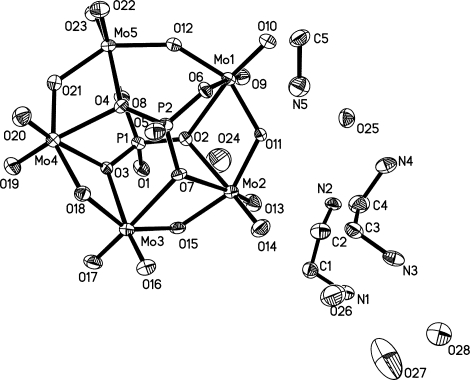
The asymmetric unit of the title compound, with atom labels and drawn at the 50% probability level for the displacement ellipsoids. H atoms have been omitted for clarity.

**Fig. 2.**
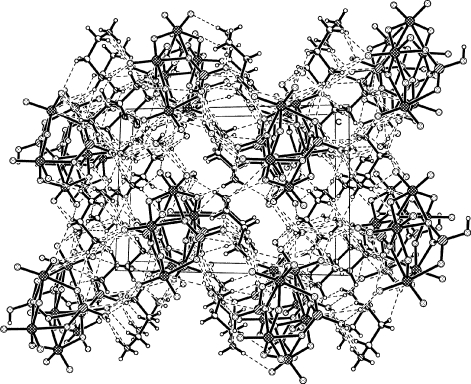
The 3-D supramolecular network construcuted by hydrogen bonds.

## supplementary crystallographic information

### Comment

Numerous polyoxometalates (POMs) have been synthesized and characterized because of their interesting structures and potential applications (Coronado *et al.*, 1998; Niu *et al.*, 2009; Ruether *et al.*, 2003). POM syntheses are usually performed under hydrothermal conditions and one or more of the reaction parameters, such as temperature, pH, stoichiometry, reaction time, can influence the reaction product. Thus the rational synthesis of POMs is still a great challenge. In a previous study, the compounds (C_2_H_10_N_2_)_2_[Mo_5_O_15_(HPO_4_)_2_] and the title compound, (C_2_H_10_N_2_)_3_(C_2_H_9_N_2_)_2_[Mo_5_O_15_(HPO_4_)_2_]^.^10H_2_O, (I), were synthesized at pH 3.0 and 5.0, respectively. Because compound (C_2_H_10_N_2_)_2_[Mo_5_O_15_(HPO_4_)_2_] has been reported in detail (Sun *et al.*, 2003), we only report the structure of compound (I).

The asymmetric unit of compound (I) contains one and a half ethylenediammonium cations, one (2-aminoethyl)ammonium cation, five lattice water molecules and one heteropolyoxometallate anion [Mo_5_O_15_(HPO_4_)_2_]^4-^. The latter is made up of five MoO_6_ octahedra sharing an edge and forming a ring which is closed by common corners of the terminal octahedron. The rings are topped on both sides by two asymmetric PO_4_ tetrahedra, sharing three corners with three MoO_6_ octahedra (Fig. 1). According to the results of valence bond calculations (Brown, 2002), both terminal oxygen atoms of the two PO_4_ tetrahedra are protonated: (bond valence sums are 1.24 for O1 and 1.29 for O5). The shortest Mo—O bond lengths are observed for terminal oxygen atoms with a mean distance of 1.708 Å, and those involving oxygen atoms of PO_4_ are the longest bond with a mean bond lengths of 2.292 Å. Mo—O bond lengths involving other oxygen atoms range from 1.917Å to 1.966 Å. All those bond lengths are similar to other reported heteropolyoxometallate anions (Sun *et al.* (2003) for [Mo_5_O_15_(HPO_4_)_2_]^4-^, and Gong *et al.* (2006) and Skibsted *et al.* (2000) for [Mo_5_O_15_(PO_4_)_2_]^6-^).

As shown in Fig. 2, lattice water molecules, the protonated ethylenediamine cations and the [Mo_5_O_15_(HPO_4_)_2_]^4-^ anions are bonded with each other via O—H···O and N—H···O hydrogen bonds to form a three-dimensional network. The geometric parameters of hydrogen bonding are listed in Table 1.

### Experimental

Compound (I) was obtained under hydrothermal conditions. (NH_4_)_6_Mo_7_O_24_˙4H_2_O (0.37 g), H_3_PO_4_ (85%, 0.2 mL) Mn(OAc)_2_^.^H_2_O (0.12 g) were added in water (15 mL). The pH value was adjusted to 5.0 by ethylenediamine, and the mixture was heated at 453 K for 5 d. Blue crystals were obtained with 15% yield (based on Mo). Elemental analysis for C_5_H_36_Mo_5_N_5_O_28_P_2_: Found: C 5.36, H 3.23%, N 6.48, P 5.79, Mo 42.07%; calcd. C 5.19, H 3.11, N 6.06, P 5.36 Mo 41.52%.

### Refinement

The H atoms attached to carbon atoms were positioned geometrically and were treated as riding on their parent atoms, with a C—H distance of 0.97 Å and *U*_iso_(H) = 1.2*U*_eq_(C). The hydrogen atoms of the water molecules, ammonium functions and O1 and O5 atoms of the phosphate groups were located in difference maps and were refined by using the 'DFIX' command with O—H = 0.85 (2) Å and N—H = 0.89 (2) Å with *U*_iso_(H) = 1.2*U*_iso_(O) and *U*_iso_(H) = 1.5*U*_iso_(O), respectively. The distance of the highest peak is 0.87 Å from O14, and the distance of the deepest hole is 1.00 Å from Mo1.

### Crystal data

**Table:**

(C_2_H_10_N_2_)_3_(C_2_H_9_N_2_)_2_[Mo_5_(HPO_4_)_2_O_15_]·10H_2_O	*Z* = 1
*M**_r_* = 2312.06	*F*(000) = 1130
Triclinic, *P*1	*D*_x_ = 2.532 Mg m^−^^3^
Hall symbol: -P 1	Mo *K*α radiation, λ = 0.71073 Å
*a* = 10.0045 (11) Å	Cell parameters from 5253 reflections
*b* = 10.6625 (12) Å	θ = 1.4–25.0°
*c* = 15.1884 (19) Å	µ = 2.23 mm^−^^1^
α = 87.405 (2)°	*T* = 298 K
β = 73.119 (1)°	Block, blue
γ = 77.978 (1)°	0.38 × 0.34 × 0.30 mm
*V* = 1516.2 (3) Å^3^	

### Data collection

**Table:**

Siemens SMART CCD area-detector diffractometer	5253 independent reflections
Radiation source: fine-focus sealed tube	4015 reflections with *I* > 2σ(*I*)
graphite	*R*_int_ = 0.033
phi and ω scans	θ_max_ = 25.0°, θ_min_ = 1.4°
Absorption correction: multi-scan (*SADABS*; Sheldrick, 1996)	*h* = −11→11
*T*_min_ = 0.485, *T*_max_ = 0.554	*k* = −12→12
7582 measured reflections	*l* = −16→18

### Refinement

**Table:**

Refinement on *F*^2^	Primary atom site location: structure-invariant direct methods
Least-squares matrix: full	Secondary atom site location: difference Fourier map
*R*[*F*^2^ > 2σ(*F*^2^)] = 0.041	Hydrogen site location: inferred from neighbouring sites
*wR*(*F*^2^) = 0.114	H-atom parameters constrained
*S* = 1.03	*w* = 1/[σ^2^(*F*_o_^2^) + (0.0528*P*)^2^ + 3.4931*P*] where *P* = (*F*_o_^2^ + 2*F*_c_^2^)/3
5253 reflections	(Δ/σ)_max_ = 0.001
396 parameters	Δρ_max_ = 1.28 e Å^−^^3^
5 restraints	Δρ_min_ = −1.06 e Å^−^^3^

### Special details

**Table:**

Geometry. All esds (except the esd in the dihedral angle between two l.s. planes) are estimated using the full covariance matrix. The cell esds are taken into account individually in the estimation of esds in distances, angles and torsion angles; correlations between esds in cell parameters are only used when they are defined by crystal symmetry. An approximate (isotropic) treatment of cell esds is used for estimating esds involving l.s. planes.
Refinement. Refinement of *F*^2^ against ALL reflections. The weighted *R*-factor wR and goodness of fit *S* are based on *F*^2^, conventional *R*-factors *R* are based on *F*, with *F* set to zero for negative *F*^2^. The threshold expression of *F*^2^ > σ(*F*^2^) is used only for calculating *R*-factors(gt) etc. and is not relevant to the choice of reflections for refinement. *R*-factors based on *F*^2^ are statistically about twice as large as those based on *F*, and *R*- factors based on ALL data will be even larger.

### Fractional atomic coordinates and isotropic or equivalent isotropic displacement parameters (Å^2^)

**Table:**

	*x*	*y*	*z*	*U*_iso_*/*U*_eq_	
Mo1	0.68911 (6)	0.98073 (6)	0.29463 (4)	0.01950 (16)	
Mo2	0.70040 (6)	0.66797 (6)	0.33717 (4)	0.01926 (16)	
Mo3	1.04111 (6)	0.51146 (6)	0.24970 (4)	0.01935 (16)	
Mo4	1.25353 (6)	0.72000 (6)	0.16806 (4)	0.01859 (16)	
Mo5	1.06247 (6)	1.02104 (6)	0.22185 (4)	0.02026 (17)	
P1	0.96988 (18)	0.79447 (17)	0.37213 (12)	0.0172 (4)	
P2	0.92311 (18)	0.77833 (17)	0.13393 (12)	0.0173 (4)	
O1	0.9504 (5)	0.7503 (5)	0.4712 (3)	0.0252 (11)	
H1F	0.9730	0.6690	0.4724	0.030*	
O2	0.8247 (5)	0.8134 (4)	0.3492 (3)	0.0194 (10)	
O3	1.0838 (4)	0.6931 (4)	0.3051 (3)	0.0165 (10)	
O4	1.0145 (5)	0.9234 (4)	0.3588 (3)	0.0204 (11)	
O5	0.9720 (5)	0.7288 (5)	0.0357 (3)	0.0253 (11)	
H5F	0.9054	0.7121	0.0184	0.030*	
O6	0.7953 (5)	0.8908 (4)	0.1520 (3)	0.0196 (10)	
O7	0.8823 (5)	0.6662 (4)	0.1989 (3)	0.0185 (10)	
O8	1.0447 (5)	0.8278 (4)	0.1577 (3)	0.0203 (10)	
O9	0.6217 (5)	1.0335 (5)	0.4066 (3)	0.0306 (12)	
O10	0.5760 (5)	1.0780 (5)	0.2421 (3)	0.0276 (12)	
O11	0.6061 (5)	0.8291 (4)	0.3003 (3)	0.0198 (10)	
O12	0.8564 (5)	1.0536 (5)	0.2577 (3)	0.0247 (11)	
O13	0.6085 (5)	0.6714 (5)	0.4513 (3)	0.0317 (13)	
O14	0.6344 (6)	0.5618 (5)	0.2884 (4)	0.0328 (13)	
O15	0.8706 (5)	0.5525 (4)	0.3514 (3)	0.0210 (11)	
O16	1.0008 (5)	0.4019 (5)	0.1883 (4)	0.0297 (12)	
O17	1.1447 (5)	0.4220 (5)	0.3112 (4)	0.0307 (12)	
O18	1.1771 (5)	0.5751 (4)	0.1505 (3)	0.0207 (11)	
O19	1.3806 (5)	0.6492 (5)	0.2178 (4)	0.0325 (13)	
O20	1.3464 (5)	0.7417 (5)	0.0556 (3)	0.0299 (12)	
O21	1.2370 (5)	0.8958 (5)	0.2099 (3)	0.0219 (11)	
O22	1.0926 (6)	1.1388 (5)	0.2799 (4)	0.0321 (13)	
O23	1.0943 (5)	1.0763 (5)	0.1119 (3)	0.0320 (13)	
O24	0.3296 (8)	0.1763 (7)	0.4162 (5)	0.076 (2)	
H24A	0.2419	0.2141	0.4404	0.091*	
H24B	0.3264	0.1133	0.3838	0.091*	
O25	0.3181 (5)	0.9219 (5)	0.3693 (3)	0.0301 (12)	
H25A	0.4032	0.8802	0.3547	0.036*	
H25B	0.2831	0.9163	0.3256	0.036*	
O26	0.4289 (7)	0.4016 (6)	0.3250 (4)	0.0511 (16)	
H26A	0.4839	0.4443	0.3370	0.061*	
H26B	0.3799	0.4467	0.2938	0.061*	
O27	0.3488 (8)	0.2320 (9)	0.1809 (5)	0.098 (3)	
H27A	0.4030	0.1839	0.2099	0.118*	
H27B	0.2619	0.2300	0.2124	0.118*	
O28	0.2690 (6)	0.2880 (6)	0.0232 (4)	0.0443 (15)	
H28A	0.3305	0.3154	0.0406	0.053*	
H28B	0.2403	0.2316	0.0625	0.053*	
C1	0.3022 (8)	0.5766 (8)	0.9035 (5)	0.0328 (18)*	
H1D	0.3735	0.5145	0.8608	0.039*	
H1E	0.3418	0.5931	0.9519	0.039*	
C2	0.2697 (9)	0.6994 (8)	0.8535 (5)	0.037 (2)*	
H2A	0.3583	0.7180	0.8138	0.045*	
H2B	0.2112	0.6877	0.8147	0.045*	
C3	0.2894 (8)	0.6276 (8)	0.4831 (6)	0.037 (2)	
H3A	0.3200	0.6701	0.4251	0.045*	
H3B	0.3715	0.5671	0.4917	0.045*	
C4	0.2356 (10)	0.7262 (8)	0.5605 (6)	0.043 (2)	
H4A	0.1792	0.6900	0.6149	0.052*	
H4B	0.3163	0.7477	0.5752	0.052*	
C5	0.5516 (8)	1.0024 (8)	0.0280 (5)	0.0295 (18)	
H5A	0.6482	0.9898	−0.0128	0.035*	
H5B	0.5289	1.0861	0.0574	0.035*	
N1	0.1738 (7)	0.5215 (6)	0.9442 (4)	0.0327 (16)	
H1A	0.1043	0.5743	0.9849	0.049*	
H1B	0.2007	0.4508	0.9739	0.049*	
H1C	0.1358	0.4996	0.9021	0.049*	
N2	0.1935 (7)	0.8119 (6)	0.9175 (4)	0.0313 (15)	
H2C	0.1776	0.8842	0.8863	0.047*	
H2D	0.2396	0.8257	0.9571	0.047*	
H2E	0.1062	0.7999	0.9507	0.047*	
N3	0.1786 (7)	0.5569 (6)	0.4790 (4)	0.0309 (15)	
H3C	0.1472	0.5167	0.5312	0.046*	
H3D	0.1649	0.5203	0.4333	0.046*	
N4	0.1468 (7)	0.8455 (6)	0.5355 (4)	0.0327 (16)	
H4C	0.1986	0.8779	0.4852	0.049*	
H4D	0.1126	0.9056	0.5792	0.049*	
H4E	0.0741	0.8210	0.5231	0.049*	
N5	0.5426 (7)	0.9020 (6)	0.0988 (4)	0.0319 (15)	
H5C	0.4558	0.9047	0.1371	0.048*	
H5D	0.6028	0.9086	0.1306	0.048*	
H5E	0.5721	0.8236	0.0713	0.048*	

### Atomic displacement parameters (Å^2^)

**Table:**

	*U*^11^	*U*^22^	*U*^33^	*U*^12^	*U*^13^	*U*^23^
Mo1	0.0177 (3)	0.0153 (3)	0.0236 (3)	−0.0010 (2)	−0.0049 (3)	0.0023 (2)
Mo2	0.0192 (3)	0.0160 (3)	0.0227 (3)	−0.0047 (3)	−0.0059 (3)	0.0036 (2)
Mo3	0.0209 (3)	0.0136 (3)	0.0234 (3)	−0.0031 (3)	−0.0066 (3)	0.0010 (2)
Mo4	0.0174 (3)	0.0174 (3)	0.0204 (3)	−0.0026 (3)	−0.0052 (2)	0.0006 (2)
Mo5	0.0202 (3)	0.0148 (3)	0.0255 (3)	−0.0046 (3)	−0.0062 (3)	0.0044 (2)
P1	0.0189 (9)	0.0153 (9)	0.0170 (9)	−0.0015 (7)	−0.0059 (7)	0.0003 (7)
P2	0.0183 (9)	0.0173 (10)	0.0165 (9)	−0.0036 (7)	−0.0055 (7)	0.0026 (7)
O1	0.029 (3)	0.020 (3)	0.023 (3)	0.001 (2)	−0.006 (2)	0.001 (2)
O2	0.017 (2)	0.018 (3)	0.023 (3)	−0.002 (2)	−0.007 (2)	0.002 (2)
O3	0.014 (2)	0.013 (2)	0.021 (2)	−0.0014 (19)	−0.0038 (19)	−0.0017 (19)
O4	0.023 (3)	0.015 (3)	0.022 (3)	−0.002 (2)	−0.005 (2)	−0.0026 (19)
O5	0.027 (3)	0.029 (3)	0.021 (3)	−0.008 (2)	−0.007 (2)	0.000 (2)
O6	0.018 (2)	0.022 (3)	0.018 (2)	0.000 (2)	−0.006 (2)	0.002 (2)
O7	0.021 (2)	0.012 (2)	0.023 (3)	−0.007 (2)	−0.005 (2)	0.0036 (19)
O8	0.017 (2)	0.018 (3)	0.027 (3)	−0.006 (2)	−0.008 (2)	0.004 (2)
O9	0.031 (3)	0.027 (3)	0.028 (3)	−0.003 (2)	−0.003 (2)	−0.003 (2)
O10	0.025 (3)	0.020 (3)	0.037 (3)	0.000 (2)	−0.012 (2)	0.005 (2)
O11	0.013 (2)	0.017 (3)	0.028 (3)	−0.001 (2)	−0.005 (2)	0.004 (2)
O12	0.022 (3)	0.017 (3)	0.035 (3)	−0.003 (2)	−0.010 (2)	0.004 (2)
O13	0.029 (3)	0.029 (3)	0.030 (3)	−0.001 (2)	−0.001 (2)	0.003 (2)
O14	0.035 (3)	0.029 (3)	0.041 (3)	−0.015 (3)	−0.017 (3)	0.005 (2)
O15	0.025 (3)	0.015 (3)	0.025 (3)	−0.007 (2)	−0.009 (2)	0.006 (2)
O16	0.030 (3)	0.021 (3)	0.039 (3)	−0.009 (2)	−0.010 (2)	0.002 (2)
O17	0.033 (3)	0.018 (3)	0.044 (3)	−0.001 (2)	−0.019 (3)	0.005 (2)
O18	0.021 (3)	0.018 (3)	0.022 (3)	−0.004 (2)	−0.005 (2)	−0.002 (2)
O19	0.025 (3)	0.027 (3)	0.047 (3)	−0.002 (2)	−0.015 (3)	0.003 (2)
O20	0.029 (3)	0.031 (3)	0.025 (3)	−0.007 (2)	0.000 (2)	−0.003 (2)
O21	0.019 (2)	0.022 (3)	0.024 (3)	−0.005 (2)	−0.005 (2)	0.000 (2)
O22	0.035 (3)	0.025 (3)	0.040 (3)	−0.010 (2)	−0.014 (3)	0.000 (2)
O23	0.034 (3)	0.030 (3)	0.030 (3)	−0.007 (3)	−0.008 (2)	0.014 (2)
O24	0.055 (5)	0.058 (5)	0.094 (6)	−0.007 (4)	0.009 (4)	−0.027 (4)
O25	0.026 (3)	0.033 (3)	0.034 (3)	−0.006 (2)	−0.013 (2)	−0.001 (2)
O26	0.050 (4)	0.044 (4)	0.063 (4)	−0.015 (3)	−0.019 (3)	0.002 (3)
O27	0.059 (5)	0.137 (9)	0.078 (6)	0.019 (5)	−0.020 (5)	0.020 (5)
O28	0.042 (3)	0.032 (4)	0.060 (4)	−0.007 (3)	−0.016 (3)	0.001 (3)
C3	0.028 (4)	0.024 (5)	0.051 (5)	0.002 (4)	−0.003 (4)	0.005 (4)
C4	0.053 (6)	0.039 (6)	0.048 (5)	−0.016 (5)	−0.027 (5)	0.013 (4)
C5	0.035 (4)	0.025 (4)	0.037 (4)	−0.016 (4)	−0.015 (4)	0.006 (3)
N1	0.044 (4)	0.026 (4)	0.025 (3)	−0.003 (3)	−0.007 (3)	0.000 (3)
N2	0.029 (4)	0.025 (4)	0.035 (4)	−0.005 (3)	−0.002 (3)	0.010 (3)
N3	0.048 (4)	0.026 (4)	0.022 (3)	−0.008 (3)	−0.015 (3)	0.006 (3)
N4	0.046 (4)	0.022 (4)	0.031 (4)	−0.010 (3)	−0.008 (3)	−0.009 (3)
N5	0.037 (4)	0.030 (4)	0.034 (4)	−0.010 (3)	−0.015 (3)	−0.003 (3)

### Geometric parameters (Å, °)

**Table:**

Mo1—O9	1.710 (5)	O24—H24B	0.8617
Mo1—O10	1.718 (5)	O25—H25A	0.8451
Mo1—O12	1.917 (5)	O25—H25B	0.8447
Mo1—O11	1.953 (5)	O26—H26A	0.8443
Mo1—O6	2.274 (4)	O26—H26B	0.8435
Mo1—O2	2.285 (4)	O27—H27A	0.8680
Mo2—O13	1.709 (5)	O27—H27B	0.8656
Mo2—O14	1.714 (5)	O28—H28A	0.8421
Mo2—O11	1.924 (4)	O28—H28B	0.8598
Mo2—O15	1.944 (5)	C1—N1	1.482 (10)
Mo2—O2	2.224 (5)	C1—C2	1.508 (10)
Mo2—O7	2.343 (4)	C1—H1D	0.9700
Mo3—O16	1.706 (5)	C1—H1E	0.9700
Mo3—O17	1.711 (5)	C2—N2	1.502 (10)
Mo3—O18	1.919 (5)	C2—H2A	0.9700
Mo3—O15	1.926 (5)	C2—H2B	0.9700
Mo3—O3	2.311 (4)	C3—N3	1.481 (10)
Mo3—O7	2.312 (4)	C3—C4	1.512 (10)
Mo4—O19	1.692 (5)	C3—H3A	0.9700
Mo4—O20	1.722 (5)	C3—H3B	0.9700
Mo4—O18	1.921 (5)	C4—N4	1.494 (10)
Mo4—O21	1.966 (5)	C4—H4A	0.9700
Mo4—O8	2.211 (4)	C4—H4B	0.9700
Mo4—O3	2.322 (4)	C5—N5	1.481 (9)
Mo5—O22	1.690 (5)	C5—C5^i^	1.525 (14)
Mo5—O23	1.709 (5)	C5—H5A	0.9700
Mo5—O21	1.932 (5)	C5—H5B	0.9700
Mo5—O12	1.933 (5)	N1—H1A	0.8953
Mo5—O4	2.253 (5)	N1—H1B	0.8934
Mo5—O8	2.380 (5)	N1—H1C	0.8952
P1—O4	1.520 (5)	N2—H2C	0.8970
P1—O1	1.526 (5)	N2—H2D	0.8910
P1—O3	1.555 (5)	N2—H2E	0.9044
P1—O2	1.561 (5)	N3—H3C	0.8896
P2—O5	1.511 (5)	N3—H3D	0.8689
P2—O6	1.528 (5)	N4—H4C	0.8901
P2—O8	1.558 (5)	N4—H4D	0.8854
P2—O7	1.560 (5)	N4—H4E	0.8918
O1—H1F	0.8500	N5—H5C	0.8890
O5—H5F	0.8360	N5—H5D	0.8888
O24—H24A	0.8641	N5—H5E	0.9043
			
O9—Mo1—O10	102.4 (2)	O6—P2—O7	109.2 (3)
O9—Mo1—O12	100.1 (2)	O8—P2—O7	109.4 (3)
O10—Mo1—O12	102.6 (2)	P1—O1—H1F	110.0
O9—Mo1—O11	100.8 (2)	P1—O2—Mo2	129.3 (3)
O10—Mo1—O11	96.4 (2)	P1—O2—Mo1	133.9 (3)
O12—Mo1—O11	147.8 (2)	Mo2—O2—Mo1	96.06 (16)
O9—Mo1—O6	173.2 (2)	P1—O3—Mo3	125.3 (2)
O10—Mo1—O6	84.3 (2)	P1—O3—Mo4	130.2 (3)
O12—Mo1—O6	79.31 (19)	Mo3—O3—Mo4	92.41 (15)
O11—Mo1—O6	77.05 (18)	P1—O4—Mo5	123.0 (3)
O9—Mo1—O2	85.7 (2)	P2—O5—H5F	112.6
O10—Mo1—O2	166.4 (2)	P2—O6—Mo1	121.6 (2)
O12—Mo1—O2	86.43 (18)	P2—O7—Mo3	125.2 (3)
O11—Mo1—O2	71.10 (17)	P2—O7—Mo2	130.2 (3)
O6—Mo1—O2	87.45 (16)	Mo3—O7—Mo2	92.82 (15)
O13—Mo2—O14	104.5 (3)	P2—O8—Mo4	129.5 (3)
O13—Mo2—O11	100.0 (2)	P2—O8—Mo5	134.9 (3)
O14—Mo2—O11	101.2 (2)	Mo4—O8—Mo5	94.63 (16)
O13—Mo2—O15	94.9 (2)	Mo2—O11—Mo1	119.7 (2)
O14—Mo2—O15	98.7 (2)	Mo1—O12—Mo5	146.5 (3)
O11—Mo2—O15	151.31 (19)	Mo3—O15—Mo2	121.2 (2)
O13—Mo2—O2	95.4 (2)	Mo3—O18—Mo4	121.2 (2)
O14—Mo2—O2	160.0 (2)	Mo5—O21—Mo4	120.0 (2)
O11—Mo2—O2	73.01 (18)	H24A—O24—H24B	105.7
O15—Mo2—O2	81.31 (18)	H25A—O25—H25B	108.9
O13—Mo2—O7	162.8 (2)	H26A—O26—H26B	109.4
O14—Mo2—O7	89.0 (2)	H27A—O27—H27B	105.9
O11—Mo2—O7	87.47 (17)	H28A—O28—H28B	106.4
O15—Mo2—O7	72.28 (17)	N1—C1—C2	112.4 (6)
O2—Mo2—O7	71.84 (16)	N1—C1—H1D	109.1
O16—Mo3—O17	104.8 (2)	C2—C1—H1D	109.1
O16—Mo3—O18	98.2 (2)	N1—C1—H1E	109.1
O17—Mo3—O18	103.4 (2)	C2—C1—H1E	109.1
O16—Mo3—O15	102.4 (2)	H1D—C1—H1E	107.9
O17—Mo3—O15	96.2 (2)	N2—C2—C1	113.0 (6)
O18—Mo3—O15	146.9 (2)	N2—C2—H2A	109.0
O16—Mo3—O3	166.0 (2)	C1—C2—H2A	109.0
O17—Mo3—O3	88.1 (2)	N2—C2—H2B	109.0
O18—Mo3—O3	73.27 (17)	C1—C2—H2B	109.0
O15—Mo3—O3	81.00 (18)	H2A—C2—H2B	107.8
O16—Mo3—O7	87.5 (2)	N3—C3—C4	112.0 (7)
O17—Mo3—O7	165.5 (2)	N3—C3—H3A	109.2
O18—Mo3—O7	82.00 (17)	C4—C3—H3A	109.2
O15—Mo3—O7	73.31 (17)	N3—C3—H3B	109.2
O3—Mo3—O7	80.46 (15)	C4—C3—H3B	109.2
O19—Mo4—O20	104.5 (3)	H3A—C3—H3B	107.9
O19—Mo4—O18	100.8 (2)	N4—C4—C3	111.8 (6)
O20—Mo4—O18	99.1 (2)	N4—C4—H4A	109.3
O19—Mo4—O21	96.6 (2)	C3—C4—H4A	109.3
O20—Mo4—O21	95.7 (2)	N4—C4—H4B	109.3
O18—Mo4—O21	153.52 (19)	C3—C4—H4B	109.3
O19—Mo4—O8	158.6 (2)	H4A—C4—H4B	107.9
O20—Mo4—O8	95.8 (2)	N5—C5—C5^i^	110.7 (8)
O18—Mo4—O8	82.59 (18)	N5—C5—H5A	109.5
O21—Mo4—O8	74.17 (18)	C5^i^—C5—H5A	109.5
O19—Mo4—O3	88.0 (2)	N5—C5—H5B	109.5
O20—Mo4—O3	166.4 (2)	C5^i^—C5—H5B	109.5
O18—Mo4—O3	72.96 (17)	H5A—C5—H5B	108.1
O21—Mo4—O3	87.89 (17)	C1—N1—H1A	113.3
O8—Mo4—O3	72.61 (16)	C1—N1—H1B	107.2
O22—Mo5—O23	103.1 (3)	H1A—N1—H1B	107.8
O22—Mo5—O21	100.0 (2)	C1—N1—H1C	113.0
O23—Mo5—O21	99.9 (2)	H1A—N1—H1C	107.1
O22—Mo5—O12	101.7 (2)	H1B—N1—H1C	108.2
O23—Mo5—O12	99.6 (2)	C2—N2—H2C	111.4
O21—Mo5—O12	146.5 (2)	C2—N2—H2D	113.7
O22—Mo5—O4	85.1 (2)	H2C—N2—H2D	108.1
O23—Mo5—O4	171.9 (2)	C2—N2—H2E	110.2
O21—Mo5—O4	78.16 (18)	H2C—N2—H2E	105.6
O12—Mo5—O4	78.63 (18)	H2D—N2—H2E	107.4
O22—Mo5—O8	168.7 (2)	C3—N3—H3C	112.0
O23—Mo5—O8	85.4 (2)	C3—N3—H3D	131.5
O21—Mo5—O8	70.87 (18)	H3C—N3—H3D	110.5
O12—Mo5—O8	83.95 (18)	C4—N4—H4C	109.0
O4—Mo5—O8	86.50 (16)	C4—N4—H4D	114.6
O4—P1—O1	111.1 (3)	H4C—N4—H4D	108.8
O4—P1—O3	109.7 (3)	C4—N4—H4E	105.9
O1—P1—O3	110.0 (3)	H4C—N4—H4E	109.6
O4—P1—O2	106.7 (3)	H4D—N4—H4E	109.0
O1—P1—O2	109.5 (3)	C5—N5—H5C	114.8
O3—P1—O2	109.8 (3)	C5—N5—H5D	108.2
O5—P2—O6	112.9 (3)	H5C—N5—H5D	109.7
O5—P2—O8	110.5 (3)	C5—N5—H5E	109.6
O6—P2—O8	106.4 (3)	H5C—N5—H5E	107.2
O5—P2—O7	108.4 (3)	H5D—N5—H5E	107.0

Symmetry codes: (i) −*x*+1, −*y*+2, −*z*.

### Hydrogen-bond geometry (Å, °)

**Table:**

*D*—H···*A*	*D*—H	H···*A*	*D*···*A*	*D*—H···*A*
N5—H5E···O20^ii^	0.90	2.66	3.075 (8)	109
N5—H5E···O28^iii^	0.90	2.01	2.796 (9)	144
N5—H5D···O10	0.89	2.45	3.069 (8)	127
N5—H5D···O6	0.89	2.01	2.846 (8)	156
N5—H5C···O21^ii^	0.89	2.17	3.046 (8)	170
N4—H4E···O1^ii^	0.89	1.93	2.806 (8)	167
N4—H4D···O12^iv^	0.89	2.65	3.357 (8)	137
N4—H4D···O22^iv^	0.89	2.60	3.099 (8)	116
N4—H4D···O4^iv^	0.89	2.08	2.907 (8)	155
N4—H4C···O25	0.89	1.92	2.803 (8)	171
N3—H3D···O17^ii^	0.87	2.25	3.117 (8)	176
N3—H3C···O15^v^	0.89	1.87	2.732 (7)	162
N2—H2E···O23^iv^	0.90	2.56	3.030 (8)	113
N2—H2E···O5^vi^	0.90	1.84	2.699 (8)	159
N2—H2D···O20^vi^	0.89	2.14	2.924 (8)	146
N2—H2C···O6^iv^	0.90	2.49	3.310 (8)	151
N2—H2C···O12^iv^	0.90	2.35	3.084 (8)	139
N1—H1C···O7^v^	0.90	2.46	3.259 (8)	149
N1—H1C···O16^v^	0.90	2.28	3.011 (8)	138
N1—H1B···O28^vii^	0.89	1.93	2.819 (8)	173
N1—H1A···O5^vi^	0.90	1.92	2.772 (8)	159
O28—H28B···O23^viii^	0.86	2.39	3.157 (8)	149
O28—H28A···O27	0.84	2.31	2.740 (10)	112
O27—H27B···O17^ii^	0.87	2.46	2.912 (10)	113
O27—H27B···O22^viii^	0.87	2.11	2.916 (10)	155
O27—H27A···O10^ix^	0.87	2.03	2.875 (9)	163
O26—H26B···O19^ii^	0.84	2.40	3.064 (8)	136
O26—H26B···O17^ii^	0.84	2.36	2.874 (8)	120
O26—H26A···O14	0.84	2.11	2.858 (8)	148
O25—H25B···O21^ii^	0.84	1.97	2.808 (7)	170
O25—H25B···O4^ii^	0.84	2.57	3.083 (7)	120
O25—H25A···O11	0.85	1.93	2.745 (7)	163
O24—H24B···O25^ix^	0.86	2.08	2.868 (9)	151
O24—H24A···O1^v^	0.86	1.97	2.795 (8)	159
O5—H5F···O28^iii^	0.84	2.02	2.845 (8)	168
O1—H1F···N3^x^	0.85	2.18	2.766 (8)	126

Symmetry codes: (ii) *x*−1, *y*, *z*; (iii) −*x*+1, −*y*+1, −*z*; (iv) −*x*+1, −*y*+2, −*z*+1; (v) −*x*+1, −*y*+1, −*z*+1; (vi) *x*−1, *y*, *z*+1; (vii) *x*, *y*, *z*+1; (viii) *x*−1, *y*−1, *z*; (ix) *x*, *y*−1, *z*; (x) *x*+1, *y*, *z*.
